# Does the epigenetic clock GrimAge predict mortality independent of genetic influences: an 18 year follow-up study in older female twin pairs

**DOI:** 10.1186/s13148-021-01112-7

**Published:** 2021-06-13

**Authors:** Föhr Tiina, Waller Katja, Viljanen Anne, Sanchez Riikka, Ollikainen Miina, Rantanen Taina, Kaprio Jaakko, Elina Sillanpää

**Affiliations:** 1 Faculty of Sport and Health Sciences, Gerontology Research Center (GEREC), University of Jyväskylä, P.O. Box 35 (VIV), 40014 Jyväskylä, Finland; 2 Faculty of Sport and Health Sciences, University of Jyväskylä, Jyväskylä, Finland; 3 Department of Public Health, University of Helsinki, Helsinki, Finland; 4 Institute for Molecular Medicine Finland (FIMM), University of Helsinki, Helsinki, Finland

**Keywords:** Biological age, DNA methylation, Epigenetic clock, Mortality, Twins

## Abstract

**Background:**

Epigenetic clocks are based on DNA methylation (DNAm). It has been suggested that these clocks are useable markers of biological aging and premature mortality. Because genetic factors explain variations in both epigenetic aging and mortality, this association could also be explained by shared genetic factors. We investigated the influence of genetic and lifestyle factors (smoking, alcohol consumption, physical activity, chronic diseases, body mass index) and education on the association of accelerated epigenetic aging with mortality using a longitudinal twin design. Utilizing a publicly available online tool, we calculated the epigenetic age using two epigenetic clocks, Horvath DNAmAge and DNAm GrimAge, in 413 Finnish twin sisters, aged 63–76 years, at the beginning of the 18-year mortality follow-up. Epigenetic age acceleration was calculated as the residuals from a linear regression model of epigenetic age estimated on chronological age (AA_Horvath_, AA_GrimAge_, respectively). Cox proportional hazard models were conducted for individuals and twin pairs.

**Results:**

The results of the individual-based analyses showed an increased mortality hazard ratio (HR) of 1.31 (CI_95_: 1.13–1.53) per one standard deviation (SD) increase in AA_GrimAge_. The results indicated no significant associations of AA_Horvath_ with mortality. Pairwise mortality analyses showed an HR of 1.50 (CI_95_: 1.02–2.20) per 1 SD increase in AA_GrimAge_. However, after adjusting for smoking, the HR attenuated substantially and was statistically non-significant (1.29; CI_95_: 0.84–1.99). Similarly, in multivariable adjusted models the HR (1.42–1.49) was non-significant. In AA_Horvath_, the non-significant HRs were lower among monozygotic pairs in comparison to dizygotic pairs, while in AA_GrimAge_ there were no systematic differences by zygosity. Further, the pairwise analysis in quartiles showed that the increased within pair difference in AA_GrimAge_ was associated with a higher all-cause mortality risk.

**Conclusions:**

In conclusion, the findings suggest that DNAm GrimAge is a strong predictor of mortality independent of genetic influences. Smoking, which is known to alter DNAm levels and is built into the DNAm GrimAge algorithm, attenuated the association between epigenetic aging and mortality risk.

**Supplementary Information:**

The online version contains supplementary material available at 10.1186/s13148-021-01112-7.

## Background

The length of the human lifespan is determined by genetic inheritance, lifestyle and environmental factors, their complex interplay, and random factors. It is generally estimated that genetic factors explain about 15–30% of the variation in lifespan. The estimates of the amount of genetic influence vary depending on the genetic ancestry and historical time of the cohort [1]. Studies with long-lived families suggest that exceptional longevity is highly heritable [2], while twin studies show that longevity seems to be only moderately heritable, and non-shared, individual environmental factors account for a majority of the variance in lifespan [3–5]. Even monozygotic (MZ) twin pairs, who share all their genetic polymorphisms and most of the early childhood and youth environment, may differ remarkably in lifespan. In these cases, within-pair differences in mortality are often caused by differences in smoking [6].

Novel measures of biological aging known as “epigenetic clocks” have been used to assess biological aging process and mortality risk. The major advantage of epigenetic clocks is that they can be utilized to estimate the progress of aging over the life course. Epigenetic clocks are based on changes in DNA methylation (DNAm, attachment of a methyl group to C-5 of cytosine base in the context of cytosine-phosphate-guanine [CpG] dinucleotide in a DNA strand) levels over time. Studies have provided evidence of age-related hypo- or hyper-methylation within specific CpG sites or islands [7], and this has laid grounds for the development of epigenetic clocks. Horvath’s algorithm was the first widely used epigenetic clock [8]. It was trained against chronological age, and therefore it has been argued that Horvath’s DNAmAge estimates may exclude CpGs, whose methylation patterns may reflect a deviation of biological age from chronological age [9]. DNAm GrimAge was subsequently developed to predict mortality [10]. It is a combination of DNAm-based surrogate biomarkers for health-related plasma proteins and smoking pack-years as well as sex and chronological age [10]. It is associated with the key “hallmarks of aging,” such as mitochondrial dysfunction and cellular senescence [11].

DNAm profiles are dependent on the nucleotide sequence of DNA strands. MZ twins in a pair have identical DNA strands, and within-pair differences in DNAm profiles are caused by various lifestyle and environmental exposures [12] as well as stochasticity. By comparing MZ to dizygotic (DZ) twin pairs, who share 50% of their polymorphic DNA sites, it is possible to differentiate genetic from environmental causes of variation in epigenetic aging and in lifestyle factors and mortality. Approximately 40–60 percent of variations in epigenetic age acceleration, depending on age and the clock utilized, are explained by additive genetic factors [13, 14].

So far, multiple studies with varying study designs and outcomes have found epigenetic age acceleration—an older DNAm age estimated by epigenetic clocks compared to chronological age—to be associated with increased mortality risk [15–19]. It has been suggested that epigenetic age predicts all-cause mortality above and beyond chronological age and traditional risk factors [20]. However, the exact mechanisms behind the association of epigenetic age acceleration and mortality are still unknown. Epigenetic age acceleration is associated with low education [21], unhealthy behavior (i.e., lifestyle risk factors of mortality), and age-related diseases [22]. Thus, these factors should be taken into account when investigating the association of epigenetic aging with mortality.

As derivation and validation of epigenetic clocks have been conducted in unrelated individuals, it is not clear whether the newer epigenetic clock DNAm GrimAge predicts lifespan irrespective of genetic influences. Previously, Christiansen et al. [17] found a stronger association of Horvath DNAm age with mortality in the oldest-old Danish twins when controlling for familial factors. The female participants of the present study are twin pairs who share sex, age, and all (MZ pairs) or half (DZ pairs) of their genetic polymorphisms and most of the intrauterine and childhood environment. This allows us to distinguish the effect of lifestyle and genetic factors on the association of epigenetic aging and mortality. The purpose of the present study was to compare two epigenetic clocks, Horvath’s DNAmAge and DNAm GrimAge, as predictors of mortality, acknowledging the effect of education and several lifestyle factors, with a subcohort of twin sisters belonging to the Finnish Twin Cohort. As GrimAge was developed to predict mortality, we hypothesize that GrimAge outperforms Horvath’s DNAmAge in mortality prediction. Because health and lifestyle related factors were taken into account in development of GrimAge, we hypothesize that GrimAge predicts mortality also independently of genetic factors. However, unhealthy lifestyle factors (such as smoking), which accelerate aging and increase disease risk, will attenuate the association between age acceleration and mortality.

## Results

### Individual-based analysis

The characteristics of the participants are presented in Table 1. Both Horvath’s DNAmAge and DNAm GrimAge predicted age in years were lower than chronological age (mean = 1.7 years lower, SD = 4.5, and mean = 8.7 years lower, SD = 3.2, respectively). Of the 413 individuals, 156 died during the study (mean follow-up time 15.8 years, range 0.2–18.3). Mortality hazard ratios (HR) with their 95% confidence intervals (CI_95_) were calculated for a 1 standard deviation (SD) increase in both Horvath’s DNAmAge and DNAmGrimAge age acceleration (AA_Horvath_, AA_GrimAge_, respectively). The mortality HR per 1 SD (3.19) increase in AA_GrimAge_ was 1.31 (CI_95_: 1.13–1.53) in model 1 adjusting for family relatedness. In model 2, including adjustment for education, all studied lifestyle factors, and the number of chronic diseases, the HR was still significant, though slightly attenuated (1.24; CI_95_: 1.02–1.51). The corresponding estimates using AA_Horvath_ were non-significant, 1.02 (CI_95_: 0.86–1.20) and 1.07 (CI_95_: 0.90–1.27), respectively (Table 2).

**Table 1:** Baseline characteristics of the participants overall and by vital status at follow-up

Characteristic	All (*N* = 413)	Alive at the end of the follow-up (*N* = 257)	Dead (*N* = 156)
Age at baseline, mean (SD), years	68.6 (3.4)	67.9 (3.2)	69.8 (3.4)
*Epigenetic clocks*			
Horvath’s DNAmAge, mean (SD), years	66.9 (5.7)	66.1 (5.6)	68.2 (5.6)
DNAm GrimAge, mean (SD), years	59.9 (4.4)	58.8 (4.0)	61.5 (4.5)
*Age acceleration (AA)*			
AA_Horvath_	− 0.03 (4.54)	− 0.09 (4.6)	0.07 (4.44)
AA_GrimAge_	− 0.05 (3.19)	− 0.40 (3.0)	0.54 (3.45)
Education, mean (SD), years	8.6 (3.0)^*^	8.9 (3.1)	8.2 (2.8)
*Cigarette smoking, n (%) of participants*			
Never smokers	362 (87.9)	230 (89.5)	133 (85.3)
Former smokers	30 (7.3)	18 (7.0)	12 (7.7)
Current smokers	20 (4.9)	9 (3.5)	11 (7.1)
*Lifetime smoking pack-years, mean (SD)*			
Former smokers	10.5 (12.7)	11.6 (14.2)	8.7 (10.4)
Current smokers	25.0 (14.7)	21.6 (4.5)	27.7 (4.8)
Body mass index, mean (SD), kg/m^2^	27.9 (4.7)	28.1 (4.9)	27.6 (4.4)
Physical activity group, mean (SD)	2.2 (1.3)	2.4 (1.3)	2.1 (1.3)
*Physical activity group, n (%) of participants*			
Mainly sedentary	117 (28.3)	68 (26.5)	49 (31.4)
Light physical activity	136 (32.9)	80 (31.1)	56 (35.9)
Moderate to vigorous physical activity	160 (38.8)	109 (42.4)	51 (32.7)
*Alcohol consumption, n (%) of participants*			
Abstainer	143 (34.6%)	76 (29.6%)	67 (42.9%)
Light drinker	197 (47.7%)	132 (51.4%)	65 (41.7%)
Moderate drinker	53 (12.8%)	36 (14.0%)	17 (10.9%)
Heavy drinker	19 (4.6%)	12 (4.7%)	7 (4.5%)
Alcohol consumption, geometric mean (SD), g/d	3.1 (5.7)	3.3 (5.5)	2.8 (5.9)
Number of chronic diseases, mean (SD)	2.0 (1.5)	1.8 (1.3)	2.3 (1.7)

^*^N = 402

Epigenetic age acceleration (AA) was calculated as the residuals from a linear regression model of epigenetic age estimate on chronological age

**Table 2:** Risks of all-cause mortality associated with a standard deviation increase in epigenetic age acceleration

	Individual analyses (*N* = 413)	Pairwise analyses among twins		
		All (*N* = 199) twin pairs	Monozygotic (*N* = 97) twin pairs	Dizygotic (*N* = 102) twin pairs
*AA*_*Horvath*_				
Model 1^∞^	1.02 (0.86–1.20)	1.05 (0.73–1.51)	0.66 (0.31–1.41)	1.22 (0.79–1.87)
Model 1 + education	1.00 (0.85–1.19)	1.00 (0.69–1.45)	0.69 (0.32–1.46)	1.14 (0.73–1.78)
Model 1 + smoking pack-years	1.01 (0.85–1.19)	0.98 (0.68–1.43)	0.67 (0.31–1.44)	1.09 (0.70–1.69)
Model 1 + BMI	1.04 (0.88–1.23)	1.05 (0.73–1.51)	0.71 (0.33–1.53)	1.22 (0.80–1.88)
Model 1 + physical activity	1.04 (0.89–1.22)	1.10 (0.76–1.60)	0.72 (0.32–1.59)	1.48 (0.91–2.41)
Model 1 + lifestyle factors^α^	1.05 (0.89–1.23)	1.02 (0.69–1.50)	0.82 (0.33–1.99)	1.32 (0.79–2.18)
Model 2^µ^	1.04 (0.88–1.23)	0.98 (0.66–1.46)	0.85 (0.35–2.08)	1.22 (0.73–2.05)
Model 2 + chronic diseases	1.07 (0.90–1.27)	0.97 (0.65–1.45)	0.85 (0.35–2.07)	1.20 (0.71–2.03)
*AA*_*GrimAge*_				
Model 1^∞^	**1.31 (1.13–1.53)**	**1.50 (1.02–2.20)**	1.37 (0.74–2.55)	1.59 (0.97–2.60)
Model 1 + education	**1.29 (1.09–1.52)**	**1.51 (1.00–2.28)**	1.39 (0.74–2.62)	1.65 (0.95–2.86)
Model 1 + smoking pack-years	**1.27 (1.05–1.54)**	1.29 (0.84–1.99)	1.16 (0.57–2.39)	1.34 (0.78–2.32)
Model 1 + BMI	**1.31 (1.13–1.52)**	**1.52 (1.03–2.25)**	1.61 (0.82–3.16)	1.59 (0.97–2.59)
Model 1 + physical activity	**1.31(1.13–1.52)**	**1.59 (1.07–2.38)**	1.83 (0.88–3.79)	**1.86 (1.07–3.21)**
Model 1 + lifestyle factors^α^	**1.31 (1.08–1.59)**	1.49 (0.93–2.38)	2.20 (0.85–5.67)	1.60 (0.86–2.97)
Model 2^µ^	**1.28 (1.05–1.55)**	1.45 (0.89–2.36)	2.58 (0.91–7.33)	1.52 (0.79–2.93)
Model 2 + chronic diseases	**1.24 (1.02–1.51)**	1.42 (0.87–2.31)	2.59 (0.91–7.38)	1.40 (0.71–2.76)

Hazard ratios and 95% confidence intervals are presented in the table. ^∞^adjusted for family relatedness ^α^ adjusted for family relatedness, smoking pack-years, BMI, physical activity and alcohol consumption ^µ^ adjusted for family relatedness, education, smoking pack-years, BMI, physical activity and alcohol consumption. BMI, body mass index. Statistically significant values are bolded. We also tested whether the estimates differed between individual twins from monozygotic and dizygotic twins and found no evidence of differences in zygosity (all adjusted *p values* 0.219 or greater for AA_Horvath_ and 0.804 or greater for AA_GrimAge_)

**Fig. 1:**
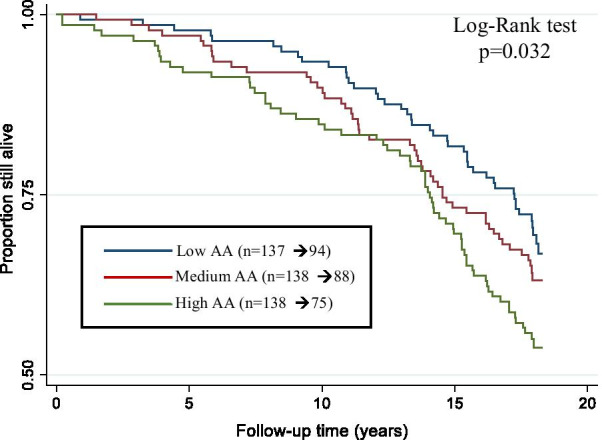
Risks of all-cause mortality according to DNAm GrimAge age acceleration (AA) tertiles

The participants were further divided into three groups according to their AA_GrimAge_ tertiles. Mean age acceleration was − 3.07 (from − 7.03 to − 1.51) in the “Slow agers” group, − 0.50 in the “Medium agers” group (from − 1.50 to 0.65), and 3.40 (from 0.65 to 13.87) in the “Fast agers” group. At baseline, the prevalence of cardiovascular diseases and hypothyroidism was highest in the fast agers group (Additional file 1: Table S1). Of the “Slow agers,” 43 of 137 (31%) died during the follow-up, and the corresponding numbers for the “Medium agers” and “Fast agers” were 50/138 (36%) and 63/138 (46%) deaths, respectively. The survival curves for mortality for these three groups are presented in Fig. 1. Compared to the “Slow agers” group, individuals in the “Fast agers” group were at higher risk of mortality; the mortality HR was 1.52 (CI_95_: 1.02–2.27). HR remained significant after adjusting for BMI and physical activity, but it was non-significant after adding other adjusting factors into the models (Additional file 1: Table S2).

### Pairwise analysis

To control for genetic and environmental factors shared within a twin pair, we performed a pairwise mortality analysis (Table 2). Of the 199 twin pairs, at least one twin died in 112 pairs during the follow-up. The mortality HR per 1 SD increase in AA Horvath was 1.05 (CI_95_: 0.73–1.51), and the non-significant estimates were systematically lower among MZ pairs in comparison to DZ pairs (test for interaction in basic model *p* = 0.228). The corresponding HR for AA_GrimAge_ was 1.50 (CI_95_: 1.02–2.20), with no systematic differences by zygosity (test for interaction in basic model *p* = 0.945). These estimates were only marginally affected when adjusted for education, body mass index (BMI), or physical activity. However, after adjusting for smoking, the HR was attenuated substantially and was statistically non-significant (1.29; CI_95_: 0.84–1.99). Similarly, in multivariable-adjusted models, the HRs (1.42–1.49) were non-significant (Table 2.). We determined twin pairs to be discordant for epigenetic aging if the within-pair difference in AA_GrimAge_ was larger than 1 SD (> 3.19 years). At the end of the follow-up, of 35 of the 60 twin pairs discordant for epigenetic aging at least one of the twins died. Moreover, the twin with the higher AA_GrimAge_ died first in 22 of these 35 twin pairs (*p* = 0.128), with no significant difference by zygosity.

**Fig. 2:**
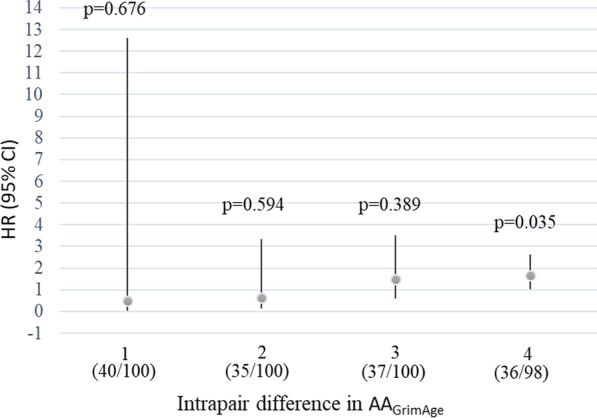
Mortality per 1 standard deviation increase in DNAm GrimAge age acceleration from the pairwise analysis. Note. Participants were divided into quartiles according to the intrapair difference in the AA_GrimAge_ (1 = the smallest difference, 4 = the greatest difference). Numbers of deaths in each of the quartiles are given in parentheses. Bars represent 95% confidence interval (CI). HR, hazard ratio; AA_GrimAge_; age acceleration

For further pairwise analysis, the twin pairs were grouped in quartiles based on intrapair difference in AA_GrimAge_, ranging from 0 to 1.12 years (no difference/minimal difference; mean = 0.54), 1.13–1.92 years (mean = 1.52), 1.94–3.70 years (mean = 2.74), and 3.73–15.85 years (great difference; mean = 6.05). The HRs per 1 SD increase in AA_GrimAge_ by quartiles were 0.50 (CI95: 0.02–12.6), 0.64 (CI95: 0.12–3.35), 1.47 (CI95: 0.61–3.51) and 1.65 (CI95: 1.04–2.63), respectively (Fig. 2). The trend was not significant (*p* = 0.56).

## Discussion

We examined the association of epigenetic age acceleration, defined by Horvath’s DNAmAge and DNAm GrimAge, with all-cause mortality within a population-based cohort of 413 Finnish twin sisters [23, 24]. Our results suggest that DNAm GrimAge outperforms Horvath’s DNAmAge in mortality risk prediction. We performed pairwise analysis in which risk for survival as a function of an epigenetic age acceleration was conducted to minimize potential pleiotropic genetic and familial influences on the association between epigenetic aging and mortality. Our genetically controlled analysis suggest that faster epigenetic aging is associated with a higher risk of mortality irrespective of genetic influences. Further, the results indicate that smoking plays an important role in the association between epigenetic aging and mortality.

In previous mortality studies, “first generation” clocks i.e. Horvath’s and Hannum’s DNAmAge have been used more widely than the quite recently developed DNAm GrimAge [18–20, 22]. We found a significant association between higher DNAm GrimAge and higher mortality risk. We investigated the association between epigenetic aging and mortality by taking into account education years and several health and lifestyle factors: smoking pack-years, BMI, physical activity, alcohol consumption, and number of chronic diseases. More precisely, in our study, a 1 SD increase in AA_GrimAge_ was significantly associated with a 31 percent increase in mortality risk, and it was only marginally affected after adjustments for education and several health and lifestyle factors. Further, our results indicated that individuals whose epigenetic aging was accelerated (“fast agers”) were at a 52 percent higher risk for mortality per SD increase in AA_GrimAge_ compared to the “slow agers.” The difference in the mortality risk between these groups was not explained by the difference in chronological age between the groups (mean age in the “slow agers” group was 68.0 years and in the “fast agers” group 68.5 years). In comparison to our findings, the few previous studies utilizing DNAm GrimAge have reported slightly higher HRs per SD increase in AA_GrimAge_; Hillary et al. reported a HR of 1.81 [16] and lifestyle risk factor-adjusted HR of 2.10 in their later study [15]. A recently published study by McCrory et al. [25] reported a HR of 2.05 per SD increase in AA_GrimAge_. In contrast to our study, which included only women, these studies included both sexes.

McCrory et al. [25] used similar methods to ours when counting the HRs per SD increase in epigenetic age acceleration. As in the present study, they reported no association between Horvath’s DNAmAge and mortality. In contrast, other previous mortality studies utilizing Horvath’s DNAmAge have counted the mortality HRs per five-year increase in DNAm age vs. chronological age. This difference in analysis strategy together with differences in sample sizes and cohorts may explain the variation of the findings of the following studies. Perna et al. [18] reported a 23 percent and Christiansen et al. [17] a 35 percent increase in mortality per five-year increase in Horvath’s DNAmAge vs. chronological age. The meta-analysis of Marioni et al. [19] used four cohorts to determine the association of epigenetic aging with mortality. A five-year higher Horvath’s DNAmAge was associated with an 11 percent higher mortality risk after adjusting for chronological age and sex. After further adjustments for several lifestyle and health factors, the mortality risk was 9 percent higher [19]. A meta-analyses by Fransquet et al. [22] indicated that each five-year increase in DNAm age was associated with an 8 to 15 percent increased risk of mortality. Studies that had assessed DNAm age with at least either the Horvath’s clock or Hannum’s clock [26] were included in this meta-analyses [22].

Our results from pairwise analysis of the twins suggest that an increased intrapair difference in AA_GrimAge_ is associated with a higher mortality risk of the co-twin with an older epigenetic age. To the best of our knowledge, no previous study has compared the association of DNAm GrimAge with mortality using a genetically controlled study design. However, Christiansen et al. [17] recently conducted a mortality analysis with Danish twins using the older Horvath’s epigenetic clock estimates. They found that the twin with a higher DNAm age had more than a twofold risk of dying first compared to his or her co-twin [17]. The use of the twin design in the present study enabled us to acknowledge the effect of the genetic and early life confounding factors in the pairwise analysis. Due to genetic factors and rearing environment, individuals may already have a DNAm age very early in life that deviates from the mean, and the pairwise analyses will to some degree control for such early-life differences. Our findings with regard to the newer GrimAge epigenetic clock indicate that the association of accelerated epigenetic aging with mortality did not differ between MZ and DZ twin pairs, and the HR risk estimates were increased rather than decreased in pairwise analysis in comparison to individual analysis. This indicates that factors other than genetics (environmental and lifestyle factors) explain the association of accelerated epigenetic aging with higher mortality.

Our results suggest that the difference between DNAm GrimAge and chronological age predicts mortality risk over and above education and several lifestyle and health factors and their combinations. However, our results indicate that smoking, which is known to change DNAm levels significantly [27], plays a significant role explaining the within-pair association of AA_GrimAge_ with mortality risk. Smoking is one of the most detrimental lifestyle factors and is associated with an increased risk for diseases [27, 28], accelerated cellular aging [29], and mortality [27, 30, 31]. In the development process of DNAm GrimAge, smoking pack-years was taken into account [10], but this does not mean that there is no need to take smoking into account in modeling. Our results suggest that in individual analysis smoking marginally attenuated the association, but was the most significant predictor of twin pair differences in age acceleration. When analyzing potential causal paths associations between age acceleration and mortality, it is important to consider smoking as a potential cause of accelerated aging. It is less likely that accelerated ageing precedes smoking as smoking is generally initiated in adolescence. Larger samples are needed to study age acceleration and mortality among never smokers.

Among the 156 participants who died by the end of the follow-up, the cause of death was accidental in six cases. Of these cases, four were accidental falls, one was exposure to heat, and one was exposure to natural forces. When we excluded these six cases from our additional mortality analysis (data not shown), the result was only marginally affected. The main reason for death of the participants in the present study was cardiovascular diseases (70 deaths, 44.9% of all deaths). Alzheimer’s disease was a reason of death in 30 cases (19.2% of all deaths) and cancer in 28 cases (17.9% of all deaths). Other reasons for death were pulmonary diseases, acute infections, and Parkinson’s disease. These are in line with common reasons of death in Finland and other economically developed countries with high life expectancies. Thus, we can generalize our results to elderly female populations in many countries. However, it must be noted that we examined only all-cause mortality. Accelerated aging have been associated with common causes of death such as cardiovascular diseases, dementia and certain cancers. It is possible that associations between epigenetic aging and mortality may vary depending on disease [22]. Due to the limited size of the FITSA cohort we were not able to conduct cause-specific mortality analyses. Analyses using large cohorts with clinical registry data about causes of death would be of high interest.

The strengths of this study were its genetically controlled twin design and the comprehensive information about participants’ lifestyle factors and education. Additionally, the participants in the present study were rather healthy at baseline. However, the number of participants was small, and the follow-up ended at the end of 2018, when over 60% of the participants were still alive. Further, the present study only included women. Previous studies have indicated that men have a higher difference between their estimated DNAm age and chronological age [19, 26]. Therefore, it is less likely that our results can be generalized to men.

## Conclusion

This study supports earlier findings showing that accelerated epigenetic aging is associated with increased mortality, and smoking plays a role by explaining this association. The present findings suggest that DNAm GrimAge is a strong predictor of mortality independent of genetic influences among female twin pairs. Further, the results indicate that this epigenetic age estimate that measures biological age and runs alongside, but not always in parallel, with chronological age may inform life expectancy predictions. Further research is needed to determine whether the results apply to men and the extent to which DNA methylation age can be used as a clinical biomarker of lifespan.

## Methods

### Participants and study design

The participants of the present study originate from The Finnish Twin Study on Aging (FITSA), which was set up to investigate the genetic and environmental effects on the disablement process in older female twins. The participants of the FITSA study were recruited from the Older Finnish Twin Cohort, which comprises all same‐sex twin pairs born before 1958 with both co‐twins alive in 1975 [32]. An invitation to participate in the FITSA study was sent to 414 female twin pairs, aged 63–76 years. The final sample of the FITSA study included 114 DZ and 103 MZ (434 individuals) twin pairs. Before the laboratory examinations during the years 2000–2001, the participants were informed about the study, and they signed a written consent form. The recruitment process of the FITSA study has been described in detail previously [23, 24]. The participants with available DNAm data are included in the present study (*N* = 413).

### DNAm age acceleration

In our previous paper, we described the generation, preprocessing, and normalization of the DNAm data [13]. Briefly, genome-wide DNAm from blood samples was determined using an Illumina EPIC BeadChip, and the data were preprocessed with the R package *minfi*. Detection *p*-values comparing the total signal for each probe to the background signal level were calculated to evaluate the quality of the samples [33]. Further analysis excluded samples of poor quality (mean detection *p* > 0.01). A single-sample Noob normalization method was used to normalize the data [34]. The epigenetic age estimates, including Horvath’s DNAmAge [8] and DNAm GrimAge [10], were produced by an online calculator (https://dnamage.genetics.ucla.edu/new). Horvath’s DNAmAge is a multi-tissue predictor of biological aging that has been developed to predict chronological age [8], while DNAm GrimAge was developed to predict lifespan [10]. Epigenetic age acceleration (the difference between chronological age and epigenetic age estimate) was calculated as the residuals from a linear regression model of epigenetic age estimate on chronological age for Horvath’s DNAmAge and DNAm GrimAge separately (AA_Horvath_, AA_GrimAge_, respectively).

### Covariates

Based on the participants’ interviews, questionnaire data, and anthropometric measurements at baseline, we obtained information on the known predictors of mortality: length of education, cigarette smoking, alcohol consumption, physical activity, chronic diseases, and BMI. Participants self-reported their education years as well as chronic diseases, which were confirmed during the medical examination conducted by a physician. Chronic diseases considered here included chronic cardiovascular, pulmonary, neurological, musculoskeletal and metabolic diseases as well as all cancers (Additional file 1: Table S1). Number of chronic diseases were calculated by adding up the diagnoses for the above diseases. Smoking status was determined based on responses to a detailed questionnaire about smoking behavior and history. The lifelong history of exposure to smoking was calculated as pack-years (equivalent to smoking 1 pack [20 cigarettes] per day for a year). Use of alcohol was measured as beverage type-specific items on frequency and quantity and converted into grams of absolute ethanol per day. For descriptive purposes, participants were further categorized as abstainers, light drinkers (3 or fewer drinks per week), moderate drinkers (more than 3 but no more than 7 drinks per week), and heavy drinkers (on average, more than a drink a day).

BMI was determined based on weight and height (weight in kilograms divided by the square of height in meters) and measured by trained research staff. Self-reported physical activity was measured using the scale developed by Grimby [35], with slight modifications. For descriptive purposes, participants were further divided into three groups of physical activity: mainly sedentary (groups 0–1), light physical activity (group 2), and moderate to vigorous physical activity (groups 3–6). The continuous seven class variable was used in the statistical analyses.

### Mortality follow-up and statistical analyses

All-cause mortality during the follow-up was analyzed. The mortality follow-up began on the date the participant participated in the laboratory measurements and the blood sampling for genome-wide DNAm analysis was conducted (during the years 2000–2001). The follow-up continued until December 31, 2018. For mortality assessment, the all-cause mortality data with exact dates of death, causes of death, and emigration from Finland were available from Statistics Finland.

### Individual-based analyses

First, we conducted a mortality analysis and calculated HRs for a 1 SD increase in AA_Horvath_ and AA_GrimAge_ with their CI_95_ for 413 individuals using the Cox proportional hazard model, clustering for family relatedness (model 1). Kaplan–Meier survival curves were tested unequal (*p* = 0.032) with the log-rank test and therefore analysis was continued in tertiles. We then adjusted the model for education years, smoking pack-years, BMI, and physical activity by adding one covariate at a time into the model. We carried out the analyses with multivariable adjustments. The model adjusted for lifestyle factors included adjustment for family relatedness, smoking pack-years, BMI, physical activity, and alcohol consumption. Model 2 was similar to the lifestyle factor-adjusted model, including an adjustment for education years. Finally, we included an adjustment for the number of chronic diseases in model 2. For further individual-based analysis, the participants were divided into three groups according to their AA_GrimAge_ tertiles, and all-cause mortality was investigated by calculating HRs during follow-up based on these tertiles.

### Pairwise analyses

Pairwise analyses were performed with the same models, but using the “strata” option for the Stata procedure *stcox* (StataIC16, StataCorp, Inc. College Station, TX, USA). This compares the hazards within pairs rather than to the overall reference category as in standard Cox regression models. Models were conducted for all twin pairs and separately for MZ pairs with an identical genomic sequence and DZ pairs sharing half of their segregating genes. The effect of zygosity was tested using the interaction term AA_GrimAge_*zygosity, comparing the fit between models with and without the interaction term. The twin pairs were further classified as discordant for epigenetic aging if the intrapair difference in AA_GrimAge_ was at least 1 SD (which corresponds to a 3.19-year difference in DNAm GrimAge). The *p*-value for the difference in whether the epigenetically “older” twin or the “younger” twin died first was derived from McNemar’s pairwise chi-square test [36]. For further pairwise analysis, the twin pairs were grouped into quartiles. The grouping was based on the deviation of the intrapair differences in AA_GrimAge_. All-cause mortality was investigated by calculating HRs during follow-up for these four groups.

## Supplementary Information

**Additional file 1.** Prevalence of chronic diseases at baseline in DNAm GrimAge age acceleration (AAGrimAge) tertiles.

## Data Availability

The mortality data used in the present study were obtained from Statistics Finland. The twin dataset used in the current study will be located in the Biobank of the Finnish Institute for Health and Welfare, Finland. All the biobanked data are publicly available for use by qualified researchers following a standardised application procedure (https://thl.fi/en/web/thl-biobank/for-researchers).

## Abbreviations

AA_GrimAge_: DNA methylation GrimAge age acceleration
AA_Horvath_: Horvath’s DNA methylation age acceleration
BMI: Body mass index
CpG: Cytosine-phosphate-guanine
DZ: Dizygotic
DNAm: DNA methylation
MZ: Monozygotic
HR: Mortality hazard ratio
SD: Standard deviation
FITSA: The Finnish Twin Study on Aging
CI_95_: 95% confidence interval

## References

[1] Ruby JG, Wright KM, Rand KA, Kermany A, Noto K, Curtis D (2018). Estimates of the heritability of human longevity are substantially inflated due to assortative mating. Genetics.

[2] van den Berg N, Roiguez-Girondo M, van Dijk IK, Mourits RJ, Mandemakers K, Janssens A (2019). Longevity defined as top 10% survivors and beyond is transmitted as a quantitative genetic trait. Nat Commun.

[3] Ljungquist B, Berg S, Lanke J, McClearn GE, Pedersen NL (1998). The effect of genetic factors for longevity: a comparison of identical and fraternal twins in the Swedish Twin Registry. J Gerontol A Biol Sci Med Sci.

[4] Herskind AM, McGue M, Holm NV, Sørensen TIA, Harvald B, Vaupel JW (1996). The heritability of human longevity: a population-based study of 2872 Danish twin pairs born 1870–1900. Hum Genet.

[5] McGue M, Vaupel JW, Holm N, Harvald B (1993). Longevity is moderately heritable in a sample of Danish twins born 1870–1880. J Gerontol.

[6] Kujala UM, Kaprio J, Koskenvuo M (2002). Modifiable risk factors as predictors of all-cause mortality: the roles of genetics and childhood environment. Am J Epidemiol.

[7] Ryan J, Wrigglesworth J, Loong J, Fransquet PD, Woods RL (2019). A systematic review and meta-analysis of environmental, lifestyle, and health factors associated with dna methylation age. J Gerontol A Biol Sci Med Sci.

[8] Horvath S (2013). DNA methylation age of human tissues and cell types. Genome Biol.

[9] Levine ME, Lu AT, Quach A, Chen BH, Assimes TL, Bandinelli S (2018). An epigenetic biomarker of aging for lifespan and healthspan. Aging (Albany NY).

[10] Lu AT, Quach A, Wilson JG, Reiner AP, Aviv A, Raj K (2019). DNA methylation GrimAge strongly predicts lifespan and healthspan. Aging (Albany, NY).

[11] Liu Z, Leung D, Thrush K, Zhao W, Ratliff S, Tanaka T (2020). Underlying features of epigenetic aging clocks in vivo and in vitro. Aging Cell.

[12] Bell JT, Spector TD (2012). DNA methylation studies using twins: what are they telling us?. Genom Biol.

[13] Kankaanpää A, Tolvanen A, Bollepalli S, Leskinen T, Kujala UM, Kaprio J (2021). Leisure-time and occupational physical activity associates differently with epigenetic aging. Med Sci Sports Exerc.

[14] Sillanpää E, Ollikainen M, Kaprio J, Wang X, Leskinen T, Kujala UM (2019). Leisure-time physical activity and DNA methylation age—a twin study. Clin Epigenet.

[15] Hillary RF, Stevenson AJ, McCartney DL, Campbell A, Walker RM, Howard DM (2020). Epigenetic measures of ageing predict the prevalence and incidence of leading causes of death and disease burden. Clin Epigenet.

[16] Hillary RF, Stevenson AJ, Cox SR, McCartney DL, Harris SE, Seeboth A (2019). An epigenetic predictor of death captures multi-modal measures of brain health. Mol Psychiatry.

[17] Christiansen L, Lenart A, Tan Q, Vaupel JW, Aviv A, McGue M (2016). DNA methylation age is associated with mortality in a longitudinal Danish twin study. Aging Cell.

[18] Perna L, Zhang Y, Mons U, Holleczek B, Saum K, Brenner H (2016). Epigenetic age acceleration predicts cancer, cardiovascular, and all-cause mortality in a German case cohort. Clin Epigenet.

[19] Marioni RE, Shah S, McRae AF, Chen BH, Colicino E, Harris SE (2015). DNA methylation age of blood predicts all-cause mortality in later life. Genome Biol.

[20] Chen BH, Marioni RE, Colicino E, Peters MJ, Ward-Caviness CK, Tsai PC (2016). DNA methylation-based measures of biological age: meta-analysis. Aging (Albany, NY).

[21] Fiorito G, McCrory C, Robinson O, Carmeli C, Ochoa Rosales C, Zhang Y (2019). Socioeconomic position, lifestyle habits and biomarkers of epigenetic aging: a multi-cohort analysis. Aging (Albany, NY).

[22] Fransquet PD, Wrigglesworth J, Woods RL, Ernst ME, Ryan J (2019). The epigenetic clock as a predictor of disease and mortality risk: a systematic review and meta-analysis. Clin Epigenet.

[23] Tiainen K, Pajala S, Sipilä S, Kaprio J, Koskenvuo M, Alen M (2007). Genetic effects in common on maximal walking speed and muscle performance in older women. Scand J Med Sci Sports.

[24] Tiainen K, Sipilä S, Alen M, Heikkinen E, Kaprio J, Koskenvuo M (2004). Heritability of maximal isometric muscle strength in older female twins. J Appl Physiol.

[25] McCrory C, Fiorito G, Hernandez B, Polidoro S, O'Halloran AM, Hever A (2021). GrimAge outperforms other epigenetic clocks in the prediction of age-related clinical phenotypes and all-cause mortality. J Gerontol A Biol Sci Med Sci.

[26] Hannum G, Guinney J, Zhao L, Zhang L, Hughes G, Sadda S (2013). Genome-wide methylation profiles reveal quantitative views of human aging rates. Mol Cell.

[27] Gao X, Jia M, Zhang Y, Breitling LP, Brenner H (2015). DNA methylation changes of whole blood cells in response to active smoking exposure in adults: a systematic review of DNA methylation studies. Clin Epigenet.

[28] U.S. Department of Health and Human Services. The Health Consequences of Smoking—50 Years of Progress: A Report of the Surgeon General. Atlanta, GA: U.S. Department of Health and Human Services, Centers for Disease Control and Prevention, National Center for Chronic Disease Prevention and Health Promotion, Office on Smoking and Health, 2014.

[29] Valdes AM, Andrew T, Gardner JP, Kimura M, Oelsner E, Cherkas LF (2005). Obesity, cigarette smoking, and telomere length in women. The Lancet.

[30] Pan A, Wang Y, Talaei M, Hu F (2015). Relation of smoking with total mortality and cardiovascular events among patients with diabetes mellitus: a meta-analysis and systematic review. Circulation.

[31] Ezzati M, Lopez AD (2003). Estimates of global mortality attributable to smoking in 2000. The Lancet.

[32] Kaprio J, Bollepalli S, Buchwald J, Iso-Markku P, Korhonen T, Kovanen V (2019). The older Finnish twin cohort—45 years of follow-up. Twin Res Hum Genet.

[33] Maksimovic J, Phipson B and Oshlack A. A cross-package Bioconductor workflow for analysing methylation array data [version 3; peer review: 4 approved]. F1000Research 2017; 5: 1281. 10.12688/f1000research.8839.1 PMC4916993 27347385

[34] Fortin JP, Triche TJ, Hansen KD (2017). Preprocessing, normalization and integration of the Illumina HumanMethylationEPIC array with minfi. Bioinformatics.

[35] Grimby G (1986). Physical activity and muscle training in the elderly. Acta Med Scand Suppl.

[36] McNemar Q (1947). Note on the sampling error of the difference between correlated proportions or percentages. Psychometrika.

