# Correction to: Does the epigenetic clock GrimAge predict mortality independent of genetic influences: an 18 year follow-up study in older female twin pairs

**DOI:** 10.1186/s13148-021-01118-1

**Published:** 2021-06-25

**Authors:** Tiina Föhr, Katja Waller, Anne Viljanen, Riikka Sanchez, Miina Ollikainen, Taina Rantanen, Jaakko Kaprio, Elina Sillanpää

**Affiliations:** 1 Faculty of Sport and Health Sciences, Gerontology Research Center (GEREC), University of Jyväskylä, P.O. Box 35 (VIV), 40014 Jyväskylä, Finland; 2 Faculty of Sport and Health Sciences, University of Jyväskylä, Jyväskylä, Finland; 3 Department of Public Health, University of Helsinki, Helsinki, Finland; 4 Institute for Molecular Medicine Finland (FIMM), University of Helsinki, Helsinki, Finland

## Correction to: Clin Epigenet (2021) 13:128 10.1186/s13148-021-01112-7

Following publication of the original article [1], the authors identified an error in the below authors’ names: the given names and family names were erroneously transposed.

The incorrect names are:

GivenName: Föhr

FamilyName: Tiina

GivenName: Waller

FamilyName: Katja

GivenName: Viljanen

FamilyName: Anne

GivenName: Sanchez

FamilyName: Riikka

GivenName: Ollikainen

FamilyName: Miina

GivenName: Rantanen

FamilyName: Taina

GivenName: Kaprio

FamilyName: Jaakko

The correct names are:

GivenName: Tiina

FamilyName: Föhr

GivenName: Katja

FamilyName: Waller

GivenName: Anne

FamilyName: Viljanen

GivenName: Riikka

FamilyName: Sanchez

GivenName: Miina

FamilyName: Ollikainen

GivenName: Taina

FamilyName: Rantanen

GivenName: Jaakko

FamilyName: Kaprio

The author group has been updated above and the original article [1] has been corrected.
