# Supplementary material for: Does the epigenetic clock GrimAge predict mortality independent of genetic influences: an 18 year follow-up study in older female twin pairs
Source: Clin Epigenetics. 2021 Jun 13;13:128. doi: 10.1186/s13148-021-01112-7 (PMC8201844; doi:10.1186/s13148-021-01112-7)
Supplement: Supplementary file 1 — Additional file 1. Prevalence of chronic diseases at baseline in DNAm GrimAge age acceleration (AAGrimAge) tertiles. [file 13148_2021_1112_MOESM1_ESM.docx]

**Table S1.** Prevalence of chronic diseases at baseline in DNAm GrimAge age acceleration (AA_GrimAge)_ tertiles.

| Disease n (%) | All | slow agers | medium agers | fast agers | P-value |
| --- | --- | --- | --- | --- | --- |
| **Cardiovascular diseases** |  |  |  |  |  |
| Coronary heart disease | 51 (12.3) | 8 (5.8) | 21 (15.2) | 22 (15.9) | 0.018 |
| Cardiac failure | 21 (5.1) | 3 (2.2) | 6 (4.3) | 12 (8.7) | 0.044 |
| Pacemaker | 4 (1.0) | 1 (0.7) | 2 (1.4) | 1 (0.7) | 0.779 |
| Hypertension | 152 (36.8) | 40 (29.2) | 50 (36.2) | 62 (44.9) | 0.025 |
| **Pulmonary diseases** |  |  |  |  |  |
| Asthma | 33 (8.0) | 12 (8.8) | 11 (8.0) | 10 (7.2) | 0.898 |
| Chronic bronchitis | 12 (2.9) | 3 (2.2) | 2 (1.4) | 7 (5.1) | 0.167 |
| Chronic obstructive lung disease | 3 (0.7) | 1 (0.7) | 0 (0.0) | 2 (1.4) | 0.366 |
| **Neurological diseases** |  |  |  |  |  |
| Multiple sclerosis | 0 | 0 | 0 | 0 | - |
| Epilepsy | 4 (1.0) | 0 (0.0) | 2 (1.4) | 2 (1.4) | 0.367 |
| Parkinson's disease | 1 (0.2) | 0 (0.0) | 1 (0.7) | 0 (0.0) | - |
| Chronic headache | 24 (5.8) | 7 (5.1) | 10 (7.2) | 7 (5.1) | 0.667 |
| **Musculoskeletal diseases** |  |  |  |  |  |
| Lumbar disk disease | 133 (32.2) | 54 (39.4) | 37 (26.8) | 42 (30.4) | 0.071 |
| Rheumatoid arthritis | 17 (4.1) | 4 (2.9) | 4 (2.9) | 9 (6.5) | 0.219 |
| Ankylosing spondylitis | 2 (0.5) | 0 (0.0) | 1 (0.7) | 1 (0.7) | 0.607 |
| Arthrosis | 246 (59.6) | 83 (60.6) | 85 (61.6) | 78 (56.5) | 0.662 |
| Fibromyalgia | 7 (1.7) | 3 (2.2) | 0 (0.0) | 4 (2.9) | 0.151 |
| **Metabolic diseases** |  |  |  |  |  |
| Hypothyroidism | 27 (6.5) | 5 (3.6) | 7 (5.1) | 15 (10.9) | 0.037 |
| Hyperthyroidism | 11 (2.7) | 1 (0.7) | 7 (5.1) | 3 (2.2) | 0.075 |
| Type 1 diabetes | 0 | 0 | 0 | 0 | - |
| Type 2 diabetes | 24 (5.8) | 4 (2.9) | 9 (6.5) | 11 (8.0) | 0.183 |
| Kidney disease | 5 (1.2) | 1 (0.7) | 3 (2.2) | 1 (0.7) | 0.448 |
| Gout | 11 (2.7) | 5 (3.6) | 4 (2.9) | 2 (1.4) | 0.504 |
| **Cancer** | 35 (8.5) | 10 (7.3) | 12 (8.7) | 13 (9.4) | 0.814 |

P-values are derived from Chi-square test.

**Table S2.** Risks of all-cause mortality according to DNAm GrimAge age acceleration (AA_GrimAge)_ tertiles. The follow-up years of female participants from the Finnish Twin Study on Aging (N=413, 63–76 years) were from 2000 to 2018.

| AA_GrimAge_ tertile | Hazard Ratio (95% CI) |
| --- | --- |
| Model 1^∞^ | (N=413) |
| “Slow agers” | 1.0 |
| “Medium agers” | 1.02 (0.68-1.52) |
| “Fast agers” | **1.52 (1.02-2.27)** |
| Model 1 + education |  |
| “Slow agers” | 1 |
| “Medium agers” | 0.99 (0.66-1.49) |
| “Fast agers” | 1.44 (0.96-2.15) |
| Model 1 + smoking pack-years |  |
| “Slow agers” | 1 |
| “Medium agers” | 1.05 (0.70-1.57) |
| “Fast agers” | 1.46 (0.97-2.22) |
| Model 1 + BMI |  |
| “Slow agers” | 1 |
| “Medium agers” | 1.03 (0.69-1.55) |
| “Fast agers” | **1.55 (1.03-2.32)** |
| Model 1 + physical activity |  |
| “Slow agers” | 1 |
| “Medium agers” | 1.01 (0.68-1.49) |
| “Fast agers” | **1.56 (1.04-2.32)** |
| Model 1 + lifestyle factors^α^ |  |
| “Slow agers” | 1 |
| “Medium agers” | 0.99 (0.66-1.47) |
| “Fast agers” | 1.50 (0.99-2.26) |
| Model 2^µ^ |  |
| “Slow agers” | 1 |
| “Medium agers” | 0.99 (0.66-1.48) |
| “Fast agers” | 1.45 (0.96-2.19) |
| Model 2 + chronic diseases |  |
| “Slow agers” | 1 |
| “Medium agers” | 0.95 (0.63-1.41) |
| “Fast agers” | 1.33 (0.88-2.03) |
| ^∞^adjusted for family relatedness  ^α^ adjusted for family relatedness, smoking pack-years, BMI, physical activity and alcohol consumption ^µ^ adjusted for family relatedness, education, smoking pack-years, BMI, physical activity and alcohol consumption. BMI, body mass index. Statistically significant values are bolded. | |
